# Sequential treatment of severe airway stenosis caused by esophageal cancer by using airway stent implantation and arterial infusion chemotherapy

**DOI:** 10.1038/s41598-022-10888-4

**Published:** 2022-04-28

**Authors:** Pengfei Xie, Shuai Wang, Wei He, Meipan Yin, Chunxia Li, Zhen Li, Xiaobing Li, Yaozhen Ma, Guang Yang, Gang Wu

**Affiliations:** 1 Department of Interventional Radiology, The First Affiliated Hospital of Zhengzhou University, No. 1, Jianshe Road, Zhengzhou, 450052 China; 2 Oncology Department, The First Affiliated Hospital of Zhengzhou University, Zhengzhou, 450052 China; 3 Department of Radiology, The Fourth Hospital of Hebei Medical University, No. 12, Jiankang Road, Shijiazhuang, 050000 Hebei China

**Keywords:** Cancer therapy, Cancer

## Abstract

The purpose of this clinical study was to investigate the efficacy and safety of airway stent implantation and transarterial infusion chemotherapy in the sequential treatment of severe airway stenosis caused by esophageal cancer. Data of patients with advanced esophageal cancer complicated by severe airway stenosis treated with airway stent implantation and transarterial infusion chemotherapy were retrospectively analyzed. Furthermore, dyspnea, clinical efficacy, adverse reactions, and survival of patients were evaluated. 71 patients were included in this study. There were 28 patients with grade III dyspnea and 43 patients with grade IV dyspnea before airway stent implantation, and 34 patients with grade I dyspnea, 35 patients with grade II dyspnea and 2 patients with grade III dyspnea after airway stent implantation. After airway stent implantation and 1–3 courses of transarterial infusion chemotherapy, 11, 41 and 19 patients had complete response, partial response and stable response respectively. Total disease control rate (DCR) and objective response rate (ORR) were 100.0% and 73.2%, respectively. During the follow-up, 32 patients died of organ failure, 24 patients died of tumor-related respiratory failure, and 10 patients died of gastrointestinal bleeding. The median survival time of all patients was 8 months, and the 1-year survival rate was 40.8%. Airway stent implantation combined with arterial infusion chemotherapy is safe and effective for sequential treatment of esophageal cancer with severe airway stenosis.

## Introduction

Esophageal cancer invasion or airway compression due to enlarged lymph node leads to stenosis in the trachea and main bronchial lumen, which in turn could result in varying degrees of dyspnea, and severe dyspnea can lead to asphyxia or even death^1,2^. In cases in which esophageal carcinoma is complicated by severe airway stenosis, radical surgical resection cannot be performed^3^. Although radiotherapy and intravenous chemotherapy are the standard treatments for advanced esophageal cancer^4^, they can not quickly alleviate the airway stenosis caused by esophageal cancer.

Airway stents can rapidly resolve airway stenosis, relieve dyspnea, and provide time and opportunity for subsequent treatment^5^. Transarterial infusion chemotherapy (TAIC) for esophageal cancer has a definite curative effect and few side effects^6–8^. Therefore, in this study, we investigated the efficacy and safety of airway stent implantation and TAIC for the treatment of severe airway stenosis caused by esophageal cancer.

## Material and methods

The clinical data of patients with esophageal cancer complicated by severe airway stenosis who were treated at our interventional treatment center from August 2013 to December 2020 were retrospectively analyzed. These data included medical, imaging, interventional procedure, and follow-up records. The data in this article showed minor overlap with those used in two previous studies (references ^7^ and ^8^). The inclusion criteria were as follows: (1) Diagnosis of esophageal cancer with large airway (trachea, main bronchus) severe stenosis based on imaging and pathological findings; (2) Intolerable dyspnea; (3) Non-feasibility of surgery as determined by consultations with thoracic surgeons; and (4) Use of airway stent implantation before arterial infusion chemotherapy for esophageal cancer. The exclusion criteria were as follows: (1) Presence of airway stenosis not caused by esophageal cancer that needed to be treated with stent implantation; (2) Treatment with TAIC without endotracheal stent implantation; and (3) Esophageal cancer with airway stenosis that was not managed by sequential therapy with airway stent implantation and TAIC. This study protocol was approved by the ethics investigation committee of The First Affiliated Hospital of Zhengzhou University. The study complied with the principles set out in the Declaration of Helsinki and was approved by the institutional ethics committee. Written informed consent was obtained from all patients.

### Airway stenting under fluoroscopy

**Figure 1:**
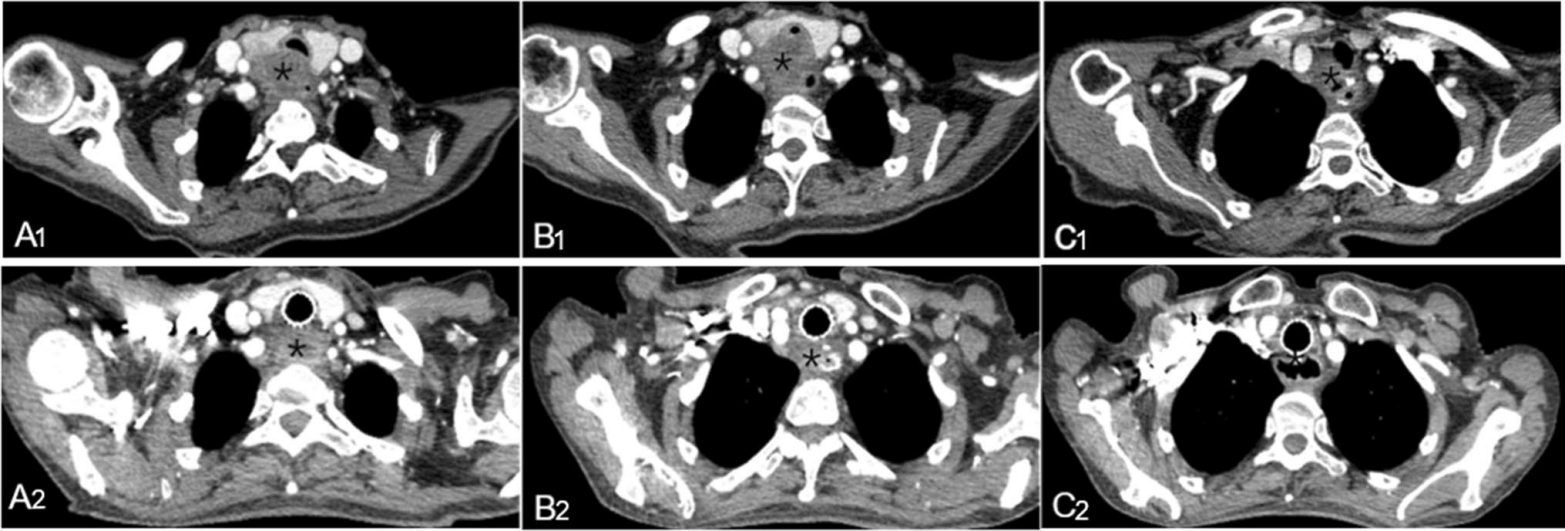
A 65-year-old female presented with dyspnea for more than one week after palliative resection of esophageal cancer. Before airway stent implantation, CT (**A1**, **B1**, **C1**) showed a soft tissue mass on the right side of the anastomosis with an irregular edge and moderate heterogeneous enhancement, and the trachea was compressed and narrowed forward. One month after TAIC, CT (**A2**, **B2**, **C2**) showed a soft tissue mass on the right side of the anastomosis, with moderate uneven enhancement, which was significantly smaller than before, and the stent shadow can be seen in the trachea. The asterisks in the figures represent the location of tumor invasion.

The degree of dyspnea was evaluated before the operation, and chest multislice spiral computed tomography (MSCT) was performed to determine the location, extent, and degree of stenosis. Furthermore, antibiotics and expectorant drugs were administered (Fig. 1). The stents were manufactured by Micro-Tech Co., Ltd., Nanjing, Jiangsu, China. If the distance between the narrow airway and glottis or that between the narrow airway and protuberance was more than 1 cm, a tubular airway stent was used. A Y-shaped airway stent is suitable for complex stenosis of the carina. In general, the length of the stent used is 1–2 cm longer than that of the stenotic segment, and the diameter of the stent is 10% larger than that of the normal trachea and main bronchus, as determined by spiral CT. Furthermore, the length of the left and right branches of the stent is shorter than that of the left and right main bronchus, so as to avoid covering the openings of the left and right upper lobes.

Diazepam 10 mg, anisodamine 10 mg and dexamethasone 10 mg were injected 20–30 min before the operation. The patient was supine on the digital subtraction angiography (DSA) table with routine oxygen inhalation, ECG monitoring, and sputum aspiration. Multi-angle fluoroscopy was used to determine the location and degree of airway stenosis. A mouth opener was placed, and a 0.035-inch hydrophilic membrane guidewire and 5F vertebral artery catheter were introduced through the mouth, throat, and trachea across the narrow segment and into the distal lumen of one lobe or the segmental bronchus. Five milliliters of 2% lidocaine was injected through the catheter for airway mucosa anesthesia to reduce irritation. If the tube airway stent was inserted, the rigid guidewire was inserted into one side of the bronchus, and the appropriate type of tracheal stent and its conveyor were introduced along the guidewire. The position of the stent was adjusted under fluoroscopy, and the stent was released accurately and quickly with the narrow segment as the center. If a Y-shaped airway stent was inserted, the AMPL guidewire was inserted into the narrow segment of the bronchus. An 8F or 9F (23 cm in length) sheath was inserted through the guidewire, and the rigid guidewire was introduced into the contralateral bronchus through the sheath. The guidewire was retained and the sheath was withdrawn. The Y-shaped airway stent and its delivery system were introduced through the two guidewires into the carina of the main bronchus or the bifurcation of the two bronchi. Fluoroscopy was used to observe the position of the stent, the degree of expansion, and airway patency. After airway stent implantation, the patients were administered anti-inflammatory and expectorant drugs as well as atomization inhalation to promote the discharge of sputum. If necessary, the patients were administered expectoration treatment under an exhaust pipe.

### TAIC

Five to seven days after stent implantation, dyspnea disappeared, or patients were able to lay flat with tolerable dyspnea under oxygen inhalation and were administered TAIC for esophageal cancer. The patients were supine on the digital subtraction angiography table. Patients were awake, and local anesthesia was applied at the right femoral artery puncture point. Femoral artery puncture was performed using a 5F arterial sheath. A 5F artery sheath was inserted into the right femoral artery by the right femoral artery puncture. A 5F Cobra catheter or vertebral artery catheter was introduced through the sheath to find the supporting artery corresponding to the lesion.

**Figure 2:**
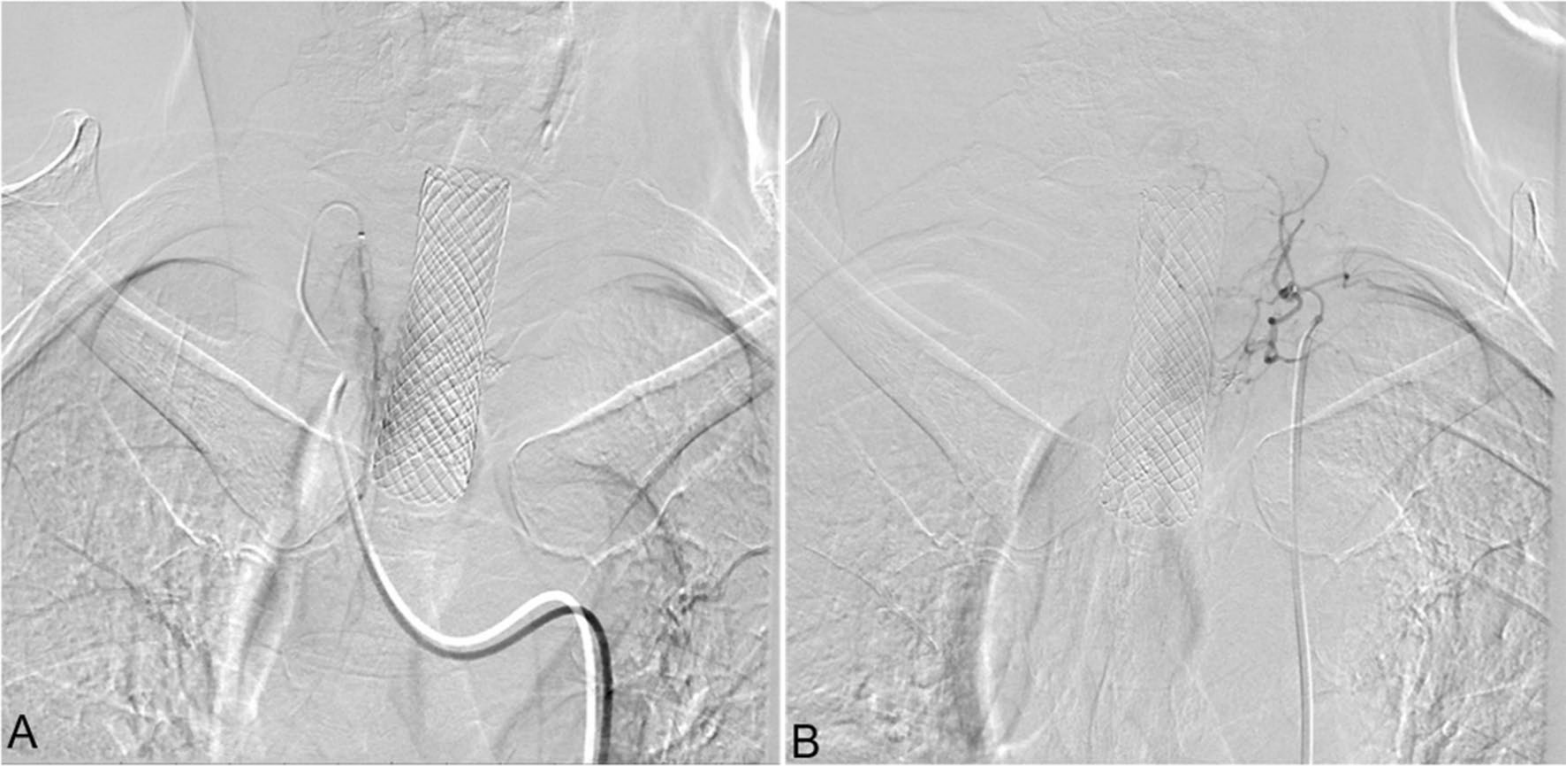
DSA showed the following findings: The right inferior thyroid artery was involved in the blood supply to the tumor, sending out many abnormal collateral vessels, and abnormally stained lesions could be seen (**A**). The left inferior thyroid artery was involved in the blood supply to the tumor and sent out many abnormal collateral vessels (**B**).

According to the patient's body surface area and physical condition, adriamycin 30–50 mg, oxaliplatin 100 mg, and raltitrexed 4 mg were administered^9^. Each chemotherapy drug was prepared as a 150–200 ml diluted solution with an appropriate compatibility solution. According to the blood supply of the target vessels, the dose of perfusion chemotherapy drugs was reasonably allocated, and a perfusion time of 15–20 min was maintained for each drug (Fig. 2). The patients were treated with antiemetic, acid suppression, and hydration therapies. Blood routine, liver and kidney function, electrolytes, and other indicators were monitored 7 days after the operation. If white blood cell and platelet counts were low, patients should receive treatment with white blood cells and platelets should be given. One month after the operation, chest CT was repeated to evaluate the curative effect (Fig. 1).

### Evaluation criteria for clinical efficacy and adverse reactions

The technical success of airway stenting is defined as accurate stent placement and satisfactory inflation, and immediate relief of dyspnea. According to the American Thoracic Association dyspnea score^10^, invasive dyspnea caused by esophageal cancer can be categorized into grades I-IV (grade I: the patient has no symptoms of dyspnea; grade II: the patient shows shortness of breath that can be relieved by rest while performing daily activities; grade III: the patient shows shortness of breath that can be relieved by oxygen while performing light activity or sitting calmly; grade IV: the patient shows shortness of breath that cannot be relieved by oxygen while sitting calmly. The improvement in respiratory function before and 5–7 days after stent implantation was evaluated. Furthermore, the clinical staging of the patients before and after treatment was evaluated according to the clinical T staging criteria of the American Joint Commission on Cancer (AJCC)^11^. Treatment efficacy for esophageal cancer was evaluated as complete response (CR), partial response (PR), stable disease (SD), and progressive disease (PD) according to the guidelines for evaluating the efficacy against solid tumors. The total objective response rate (ORR) was defined as CR + PR, and the disease control rate (DCR) was defined as CR + PR + SD^12,13^.

If CR was achieved, radiotherapy or surgery could be considered. If PR or SD was achieved, pulse perfusion chemotherapy could be considered again. If PD was detected, other palliative treatments could be considered. Further, adverse reactions to chemotherapy drugs were recorded. The toxicity and side effects of the chemotherapy drugs were evaluated according to National Cancer Institute Common Terminology Criteria for Adverse Events (NCI-CTCAE, version 4.0) and classification of anticancer-drug toxicity (0–IV).

### Statistical analysis

All data were analyzed using GraphPad Prism 5 software. The quantitative data were analyzed with independent two-sample t-tests, and the results were expressed as mean ± standard deviation values. Chi-square test was used for classification data, and a *P-*value of < 0.05 was considered statistically significant.

## Results

Seventy-one patients having esophageal carcinoma with severe airway stenosis were included. The patients included 48 men and 23 women (age range: 41–83 years; mean age: 63.5 ± 8.6 years). The locations of the esophageal cancer lesions were as follows: upper-segment lesions, 29 cases; middle-segment lesions, seven cases; lower-segment lesions, three cases; and tumor recurrence in the anastomotic region after esophageal cancer resection, 32 cases. 71 patients were treated with systemic chemotherapy after confirmation of esophageal cancer. Among the 71 patients, 45 had main airway stenosis, 13 had carina stenosis, 12 had left main branch stenosis, and one had right main bronchial stenosis. Four patients had left main bronchial stenosis with atelectasis. There were 17 cases of esophageal cancer complicated by esophageal fistula, including 12 cases of esophagotracheal fistula, three cases of esophageal mediastinal fistula, and two cases of esophagogastric anastomotic neck fistula (Table 1).

**Table 1:** Patient characteristics.

	Number of cases
Patients	71
Males	48 (67.6%)
Mean age, years	63.5 ± 8.6
**Esophageal cancer location**	
Upper esophagus	29 (40.8%)
Middle esophagus	7 (9.9%)
Lower esophagus	3 (4.2%)
Esophageal anastomosis	32 (45.1%)
**Location of airway stricture**	
Carina of main trachea	13 (18.3%)
Right-main bronchus	1 (1.4%)
Left-main bronchus	12 (16.9%)
Main trachea	45 (63.4%)
**Types of esophageal fistula**	
Esophageal airway	12 (70.6%)
Esophageal neck	2 (2.8%)
Esophageal mediastinum	3 (4.2%)
**cTNM stage of the patients**	
IVA	67 (94.4%)
IVB	4 (5.6%)

All 71 patients underwent airway stent implantation and TAIC. A total of 71 airway stents, including 43 tubular stents and 28 Y-shaped stents, were implanted in the 71 patients. Four patients with esophageal fistulas received a covered endotracheal stent, which addressed the airway stenosis and blocked the fistula. One patient with an esophageal fistula received a covered esophageal stent, while 12 patients with esophageal fistulas received a nutrition tube.

In 71 patients, 1–4 feeding arteries of the tumor were identified and perfused with chemotherapy drugs, including the bilateral inferior thyroid artery (19 cases), unilateral inferior thyroid artery (15 cases), the bilateral bronchial artery (31 cases), the unilateral bronchial artery (33 cases), the proper esophageal artery (six cases), the right gastroepiploic artery (17 cases), the thyroid artery (nine cases), the gastroduodenal artery (two cases), the phrenic artery (one case), the right internal thoracic artery (four cases), and the right gastric artery (two cases). Intraoperatively, a microcatheter was used for super-selective intubation in 69 cases to protect blood vessels and to avoid injury to the spinal arteries and drug reflux. Fifty-seven, 12, and two patients received one, two, and three courses of TAIC, respectively.

### Evaluation of clinical efficacy

Dyspnea was relieved in all 71 patients after airway stent implantation. Four patients with left main bronchus stenosis and atelectasis were treated with Y-shaped metal stents, respectively. CT after airway stent implantation showed that the lungs were completely open in two cases and partially open in one case. The ATA classification of dyspnea showed 28 cases of grade III dyspnea and 43 cases of grade IV dyspnea preoperatively. At 5–7 days after airway stent implantation, oxygen saturation was more than 95% without oxygen inhalation, including 34 cases of grade I dyspnea, 35 cases of grade II dyspnea, and two cases of grade III dyspnea. Dyspnea significantly improved in comparison with that before stent implantation. The technical success rate of airway stenting was 100%.

The clinical stage of all 71 cases before treatment was T4b. After 1–3 courses of treatment, follow-up assessments showed that the clinical stages were T1 (five cases), T2 (10 cases), T3 (13 cases), and T4b (43 cases). Thus, the clinical stage decreased significantly after treatment (Table 2).

**Table 2:** Clinical classification before and after transarterial infusion chemotherapy.

Classification	Before treatment	After the first course	After the second course	After the third course
n	71	71	14	2
T1	0	1	3	1
T2	0	12**	2	1
T3	0	13***	4	0
T4b	71	45***	5	0

***p < .0001; **p < .005; *p < .05.

After the first course of TAIC, eight cases showed CR; 44 cases showed PR; 19 cases showed SD; and the ORR was 73.2%. Fourteen patients received a second course of TAIC for esophageal cancer. After the second course, two cases showed CR; 10 cases showed PR; two cases showed SD; and the ORR was 85.7%. Two patients received a third course of TAIC for esophageal cancer. After the third course, both patients showed CR. After 1–3 courses of treatment, 11 cases showed CR; 41 cases showed PR; 19 cases showed SD; and the ORR was 73.2%, while the DCR was 100% (Table 3).

**Table 3:** Clinical efficacy of transarterial infusion chemotherapy.

Classification	After the first course	After the second course	After the third course	Follow up
n	71	14	2	71
Complete response	8 (11.3%)	2 (14.3%)	2 (100%)	11 (15.5%)
Partial response	44 (62.0%)	10 (71.4%)	0	41 (57.7%)
Stable disease	19 (26.8%)	2 (14.3%)	0	19 (26.8%)
Overall response rate	52 (73.2%)	12 (85.7%)	2 (100.0%)	52 (73.2%)
Disease control rate	71 (100%)	14 (100%)	2 (100%)	71 (100%)

Overall response rate = complete response + partial response.

Disease control rate = complete response + partial response + stable disease.

### Complications

Grade I-III adverse reactions were observed after TAIC for esophageal cancer, and these were relieved within a short time after symptomatic treatment (Table 4).

**Table 4:** Adverse reactions after transarterial infusion chemotherapy.

	I	II	III
Reduction in white blood cell count	2	0	2
Thrombocytopenia	3	0	0
Vomiting	21	4	0
Fever	1	1	2

### Follow-up

**Figure 3:**
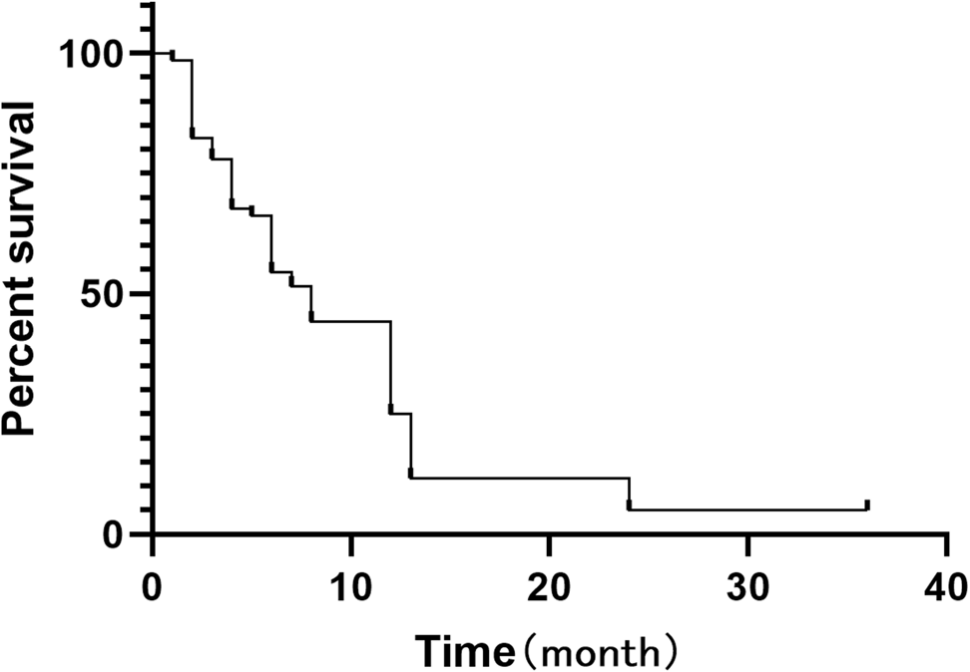
Survival curve of the patients.

The patients were followed up over telephone interviews or outpatient visits, and the median follow-up duration was 8 months. Thirty-two patients died of systemic organ failure, and 24 patients died of tumor-related respiratory failure. Ten patients died of gastrointestinal bleeding. Among the five surviving patients, two patients received intermittent chemoradiotherapy after TAIC. One patient currently has no dyspnea symptoms with a survival time of 3 years. One patient developed dyspnea after discharge. Because of stent restenosis, the patient underwent airway stent implantation in another hospital and currently has no dyspnea, with a survival time of 1 year. One patient did not receive any other treatment after discharge, with a survival time of 1 year. One patient underwent immunotherapy after TAIC with regular review and currently has a stable lesion, with a survival time of 3 years. The median survival time was 8 months, and the 1-year survival rate was 40.8% (Fig. 3).

## Discussion

Airway invasion of advanced esophageal cancer is a progressive process, and patients often show severe dyspnea that is very difficult to deal with clinically. Without active treatment, the survival time of esophageal cancer patients with airway stenosis is only 1–2 months, and the quality of life of these patients during this period is very poor, with most of them often dying of asphyxia.

The general condition of advanced esophageal cancer patients with severe airway stenosis is poor, and they often show difficulty in tolerating the toxicity and side effects of intravenous chemotherapy drugs^14^. In esophageal cancer complicated with severe airway stenosis, radiotherapy shows a limited curative effect, and radiation pneumonia can frequently appear after radiotherapy, aggravating the symptoms of dyspnea^15,16^. In addition, some local bronchoscope-assisted treatment methods, such as ablation, high-frequency electric knife, and mechanical debridement of airway, show a good curative effect in patients with mild to moderate dyspnea, but often lead to the recurrence of malignant airway stenosis symptoms. Thus, these treatment methods are not suitable for intolerable malignant airway stenosis^2,17^.

Treatment of intolerable dyspnea in esophageal cancer with airway stenosis is an urgent issue. The airway stent can immediately relieve dyspnea immediately and has become an important means to solve benign and malignant tracheal stenosis^18,19^. The advantages of airway stenting by tracheoscopy are that the procedure is easy to perform and can be conducted at the bedside. For patients with severe airway stenosis, a fiberoptic bronchoscope cannot pass through the narrow part, but a slender catheter and guidewire can be guided into the trachea under fluoroscopy, which can easily cross the narrow section and release the stent under guidance of guide wire, with less pain^18^. In this study, 71 patients showing esophageal cancer with airway stenosis experienced significant dyspnea relief after airway stent implantation, including four cases of left main bronchial stenosis with atelectasis and three cases of atelectasis after CT-confirmed complete remission. Although airway stents can improve the quality of life of patients with esophageal cancer complicated with airway stenosis, they may result in complications such as stent restenosis, stent displacement, and stent rupture. Especially for malignant airway stenosis, the tumor tissue continues to grow, which may invade the tracheal stent and accelerate the occurrence of stent restenosis^20^. Moreover, although airway stenting is only a palliative measure, it only alleviates or relieves airway stenosis physically and fails to treat the primary disease, creating favorable conditions for follow-up treatment.

In comparison with traditional intravenous chemotherapy, TAIC shows stronger local efficacy and fewer systemic side effects. Under the premise of solving dyspnea, early TAIC can not only reduce and avoid the formation of tracheal stent restenosis, but also reduce the tumor size and degrade the tumor stage. We have previously^7^ reported that among 75 patients with advanced esophageal cancer, 20 underwent airway stenting due to severe dyspnea. After 1–3 cycles of treatment, the clinical stages of the patients were reduced. Thirteen patients with mild dyspnea did not undergo airway stenting. After TAIC, the tumor shrank, the symptoms of tracheal compression or invasion were relieved, and dyspnea was improved. In the present study, the dyspnea symptoms of 71 patients with esophageal cancer and moderate-to-severe airway stenosis were greatly relieved after the placement of airway metal stents. After 1–3 courses of TAIC, the DCR was 100%.

The degree of dyspnea in patients with esophageal cancer complicated with airway stenosis should be reasonably evaluated. If the patients cannot tolerate it, airway stenting should be performed immediately. Selection of the appropriate airway stent before tracheal stent implantation, placement of the airway accurately and quickly under intraoperative fluoroscopy, and prompt postoperative evaluation of the degree of dyspnea relief are extremely important. We believe that an accurate and comprehensive search for the feeding artery of esophageal cancer is the premise for achieving a good curative effect of TAIC. The search for the feeding artery of esophageal cancer should be performed according to the general rules of feeding vessels of esophageal cancer and DSA findings, and the application of a microcatheter is also very necessary^8^. Although serious complications of arterial infusion chemotherapy for esophageal cancer are rare, the serious consequences of such complications necessitate close attention. In particular, for patients with advanced age, low immunity, repeated treatment and signs of esophageal perforation, the choice of drugs and dosage of TAIC should be performed with care. Airway stenting with sequential TAIC for esophageal cancer is a tumor reduction surgery, unlike radical surgery, so transformation therapy is very important.

The limitation of this study was that this was a single-center retrospective analysis with selection bias, so we hope to conduct a multi-center, large sample prospective study to obtain more objective and sufficient data in the future.

In summary, for patients with esophageal cancer complicated with malignant airway stenosis, airway stents can be placed first to alleviate their intolerance of dyspnea and provide opportunities for additional follow-up treatment. In combination with TAIC, sequential treatment of esophageal cancer complicated with severe airway stenosis is safe and effective, which is worthy of clinical application.
